# Serotonin's Role in Inflammatory Signaling Pathway Modulation for Colon Cancer Suppression

**DOI:** 10.7759/cureus.66040

**Published:** 2024-08-02

**Authors:** Supreeta Maheshwarla Saravanan, Lavanya Prathap, Jabir Padathpeedika Khalid, Taniya Mary Martin, Meenakshi S Kishore Kumar

**Affiliations:** 1 Department of Anatomy, Biomedical Research Unit and Laboratory Animal Centre (BRULAC) Saveetha Dental College and Hospital, Saveetha Institute of Medical and Technical Sciences, Saveetha University, Chennai, IND; 2 Department of Physiology, Saveetha Medical College and Hospital, Saveetha Institute of Medical and Technical Sciences, Saveetha University, Chennai, IND

**Keywords:** cancer inhibition, anti-inflammatory activity, anti-inflammatory effect, serotonin, mismatch repair, intermediate risk, colon cancer, adjuvant chemotherapy

## Abstract

Background

Neurons can be effectively regulated by serotonin and dopamine. Their role in anti-inflammatory pathways opens new doors for therapeutic research, particularly in chemotherapeutics. The present study investigated serotonin's role in suppressing inflammation and its potential anticancer effects in KERATIN-forming tumor cell line HeLa cells (KB cells).

Methods - in vitro and in silico analysis

The study delved further into the molecular mechanisms by assessing the expression levels of key markers involved in inflammation and cancer progression, such as B-cell leukemia/lymphoma 2 protein (BCl-2), tumor necrosis factor-alpha (TNF-α) and Interleukin-6 (IL-6) using Real-time reverse-transcriptase-polymerase chain reaction at concentrations below the IC_50_ (50 and 100 µg/ml). The binding capability of serotonin (CID 5202) with glycoform of human interleukin 6 (PDB: 7NXZ) was analyzed with the help of Schrodinger molecular suites.

Results

The findings showcased serotonin's potent growth inhibition in KB cells, with an IC_50_ value of 225±3.1µg/ml. Additionally, it demonstrated a multifaceted impact by downregulating the expression of BCl-2, TNF-α, and IL-6, pivotal factors in cancer cell survival and inflammation regulation. The docking score was - 5.65 (kcal/mol) between serotonin and glycoform of Human Interleukin 6. It is bound with ASN 143 by two hydrogen bonds. Thus, molecular docking analysis showed an efficient bounding pattern. The research findings indicate that serotonin successfully blocks NF-κB pathways in KB cells, underscoring its therapeutic promise against colon cancer and offering vital information for additional clinical investigation.

Conclusion

According to the study's conclusion, serotonin has a remarkable anticancer potential by effectively blocking NF-κB B pathways in KB cells, revealing its promising potential as a therapeutic agent against colon cancer. These comprehensive findings offer significant insights into serotonin's intricate molecular interactions and its profound impact on cancer-related signaling pathways, paving the way for further exploration and potential clinical applications in cancer treatment strategies.

## Introduction

Despite having very different molecular structures, dopamine and serotonin are neurotransmitters that have a tremendous impact on behavior and brain function. These structural differences impact how they interact with brain enzymes and receptors, which in turn shapes the physiological effects. Both neurotransmitters are essential for mood, thought, and behavior regulation [1-3]. Knowledge of the precise roles these substances play in health and illness, as well as creating focused therapy strategies for a range of neuropsychiatric conditions, requires an understanding of the complex interplay between their chemical structures and biological activities, including cytokine regulation [4].

Dopamine receptors are responsible for producing and modulating the effects of cytokines. According to research, depending on the situation, dopamine may either increase or decrease cytokine production [5]. For example, in response to inflammatory stimuli, dopamine has been demonstrated to increase the production of pro-inflammatory cytokines like interleukin-6 (IL-6) and tumor necrosis factor-alpha (TNF-α) [6]. However, under some circumstances, dopamine can also have anti-inflammatory effects by preventing the production of pro-inflammatory cytokines. On the other hand, serotonin, which is mostly produced by brain neurons and enterochromaffin cells in the gastrointestinal system, also affects the production of cytokines by binding to receptors on immune cells. It has been shown that serotonin primarily reduces inflammation by inhibiting the synthesis of pro-inflammatory cytokines such as TNF-α, IL-6, and interleukin-1β (IL-1β) [7]. Furthermore, it has been suggested that serotonin stimulates the production of cytokines that reduce inflammation, including interleukin-10 (IL-10). Dopamine and serotonin have differing impacts on the generation of cytokines, which emphasizes their involvement in controlling inflammation and immunological responses [7]. Deciphering the complicated link between neurotransmission and immunological function requires an understanding of these intricate interactions, which may also provide a new perspective for the development of therapeutic approaches aimed at treating neuroimmune illnesses [8, 9].

Dopamine and serotonin have different effects on anti-cancer activity at the cellular level, which is consistent with their different functions in downregulating cellular processes that are pertinent to the onset and spread of cancer [10]. Dopamine is mostly produced by neurons, but it may also be produced by different immune cells, certain cancers, and other tissues. Depending on the situation, dopamine may have pro- or anti-cancer effects [11]. Dopamine can, however, also have anti-cancer effects by stopping the growth of some cancer cell types and causing them to undergo apoptosis [12]. Furthermore, dopamine has been demonstrated to augment the immune response to tumors by stimulating the activation and function of cytotoxic immune cells, including natural killer cells and T lymphocytes. On the other hand, serotonin, which is mostly produced by brain neurons and enterochromaffin cells in the gastrointestinal system, has been linked mainly to the promotion of anti-cancer actions [13]. Through its modulation of many signaling pathways involved in cell cycle control and death, serotonin can decrease the proliferation, migration, and invasion of cancer cells. Serotonin has also been demonstrated to support immune surveillance against cancer cells and inhibit tumor angiogenesis [5-6, 8]. Clarifying these processes might provide important information for the creation of cutting-edge therapeutic approaches for the prevention and treatment of cancer. Serotonin has also been demonstrated to support immune surveillance against cancer. In our previous study, we showed that dopamine inhibited the pro-inflammatory pathways through the attenuation of Ak strain transforming (Akt) signaling axis in KERATIN-forming tumor cell line HeLa (KB) cell lines [14]. The present study aimed to analyze the effect of serotonin on KB cells based on Akt signaling members.

## Materials and methods

Cell viability assay

All the chemicals used in the present study were of molecular biological grade. The viability of KB cells, sourced from The National Centre for Cell Science (NCCS), Pune, India, was assessed with serotonin using the 3-(4,5-dimethlylthiazol-2-yl)-2,5-diphenyltetrazolium bromide (MTT) method. Briefly, the KB cells were cultured at a density of 10^3 cells per well in Dulbecco's Modified Eagle Medium (DMEM) supplemented with 10% fetal bovine serum (FBS) and 1% antibiotic solution (penicillin-streptomycin), followed by incubation at 37°C with 5% carbon dioxide (CO_2_) for 24 hours to ensure cell adherence. The experiment commenced when cells reached 80% confluency, treating them with varying concentrations of serotonin (50, 100, 250, 400, and 500 µg/ ml) dissolved in a maximum of 0.1% dimethyl sulfoxide (DMSO). Cell viability was evaluated after 48 hours and compared to untreated cells to ascertain the IC_50_ using the probit method [15, 16]. Furthermore, a protein denaturation assay was conducted based on established protocols [17].

Reverse transcriptase polymerase chain (RT-PCR) reaction

After 48 hours of exposure to serotonin, KB cells underwent RNA isolation using the TRIzol reagent (Takara Bio, Dalian, China) to extract total RNA. Subsequently, reverse transcription of the RNA into cDNA was performed utilizing the PrimeScript RT Reagent Kit (Takara Bio, Dalian, China). Amplification of the resultant cDNA was carried out using the Mx3005P Real-Time PCR System (Agilent, Santa Clara, California), following the manufacturer's protocols included in the Q-PCR experiment. To assess relative mRNA expression levels, normalization was conducted using GAPDH RNA through the 2CT method. The primers utilized in this experiment were sourced from Invitrogen, based in Shanghai, China [18].

Molecular docking analysis

The chemical structure of serotonin (CID 5202) from PubChem and the structure of the synthetic glycoform of Human Interleukin 6. (PDB: 7NXZ) were obtained and retrieved for analysis. The Schrodinger (Paid version) software (Maestro molecular Interface (version 11v5) of Schrödinger suite) was employed to prepare the different Epik states (a specific protonation state of the ligand and the protein, Epik could be determined by the software itself) and optimize the ligand for subsequent docking studies. The docking procedures were conducted using the extra precision method (XP), and Glide scores were calculated in units of kilocalories per mole (kcal/mol) to assess the binding interactions and affinity between serotonin and the NF-κB structure [19].

Protein denaturation inhibition assay

The albumin denaturation inhibition assay is a versatile assay for determining the bioavailability, dosage, dynamics and physiological preference of the given drug. Hence, this assay is useful to analyze the immunology-related interactions of the given drug. Ibuprofen is a well immunologically well-characterized drug that extensively binds with the albumins. Its binding nature needs to be documented in research analysis on the less-characterized compounds, including various pharmacological preferences. A 5 mL reaction mixture containing 0.2 ml of fresh egg albumin and 0.05 ml of different concentrations of serotonin and ibuprofen (0.1, 1, 5, 10, 25, 50, and 100 µg/ml, respectively) was prepared. The volume was made up to 5 ml with phosphate buffer (pH 7.4, 0.1 mM) and kept for 30 minutes at 40°C. Then, it was kept at 70°C for five minutes. The mixture was cooled and spectroscopically read at 680 nm [20].

Statistical evaluation

The significant (p) value (p<0.05) was confirmed using one-way/two-way ANOVA (Bonferroni post hoc test or Newmann Keuls post hoc test).

## Results

In this study, serotonin decreased TNF-α, NF-β, and p38 expression but enhanced Bax on KB cells.

Inhibition of protein denaturation using fresh egg albumin

**Figure 1:**
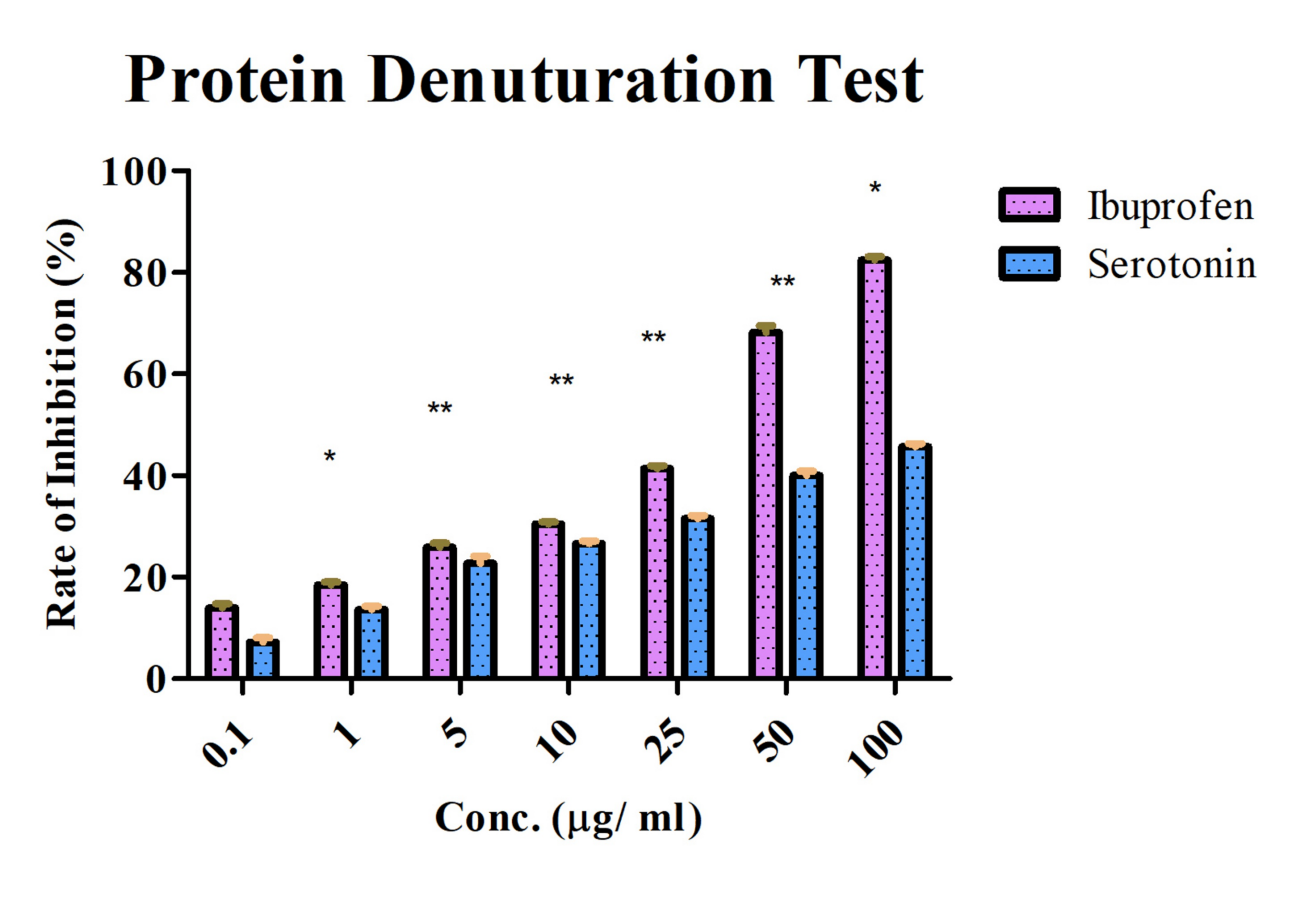
Inhibition of protein denaturation using fresh egg albumin Comparison between serotonin and Ibuprofen. The results were statistically significant. Two-way ANOVA, Bonferroni post hoc test (* represents p<0.05, ** represents p<0.005, *** represents p<0.001) Image credit: author Lavanya Prathap

The protein denaturation inhibitory activity of serotonin was evaluated using fresh egg albumin. The Ibuprofen was used as a positive control. The results showed that Ibuprofen and serotonin exhibited comparatively similar activity in the test (they showed LC_50 _as 96.25 and 100.23 µg/ ml, respectively). Both of them showed dose-dependent activity, and the values were statistically significant (Figure 1). At higher concentrations, serotonin showed slightly higher activity than Ibuprofen for one of the following reasons: dosage, molecular mechanism, stability, pharmacokinetics, and so on. The results showed the inhibitory activity of the serotonin.

Serotonin and KB cell viability

**Figure 2:**
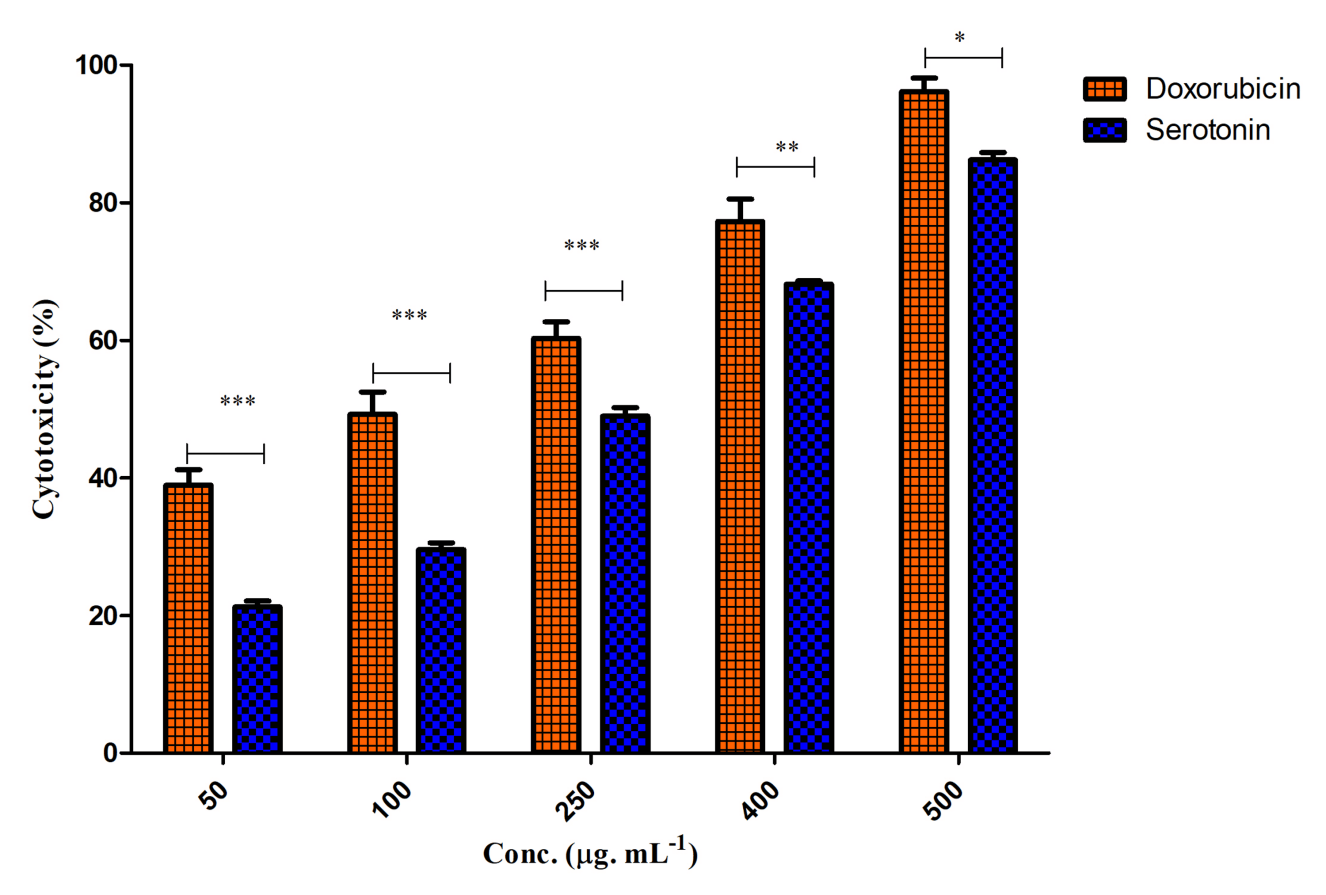
Effect of doxorubicin and serotonin on KB cells Two-way ANOVA, Bonferroni post hoc test (* represents p<0.05, ** represents p<0.005, *** represents p<0.001) Image credit: author Lavanya Prathap

MTT assay was used to analyze the cytotoxic effect of serotonin on KB cells. The findings revealed that serotonin showed cytotoxicity in a concentration-dependent manner on KB cells (Figure 2). Doxorubicin and serotonin showed 129.71±1.56 and 255±3.1 µg/ml, respectively, as their IC_50_. Pro- and anti-inflammatory gene expression levels were 50 and 100 ng/ml, respectively.

Effect of serotonin on KB cells

**Figure 3:**
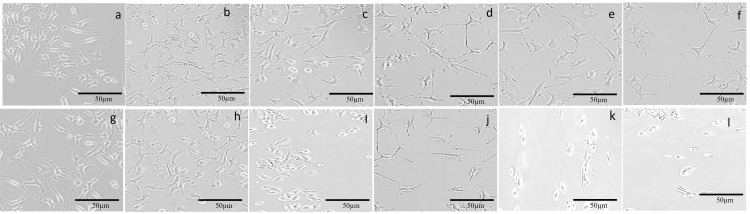
Effect of doxorubicin and serotonin on KB cells a, g) control (without drug); b-f) 50, 100, 250, 400, and 500 µg/ ml of doxorubicin, respectively; h-l) 50, 100, 250, 400, and 500 µg/ ml of serotonin, respectively Credit: Lavanya Prathap

Cytotoxic effects of serotonin were determined using the MTT assay. Doxorubicin was used as a standard control for cytotoxicity analysis. Different concentrations (50, 100, 250, 400, and 500 µg/ ml) of serotonin were used for the study. The results showed that both of the drugs, doxorubicin and serotonin, exhibited dose-dependent cytotoxicity with the KB cells (Figure 3). When compared to the control, the serotonin concentration (100-400 µg/ ml) employed in this investigation had the greatest ability to prevent cell growth. IC_50_ values of doxorubicin and serotonin was calculated as 318±1.2 and 325±2.1 µg/ ml, respectively.

Serotonin-inhibited BCl-2 in KB cells

**Figure 4:**
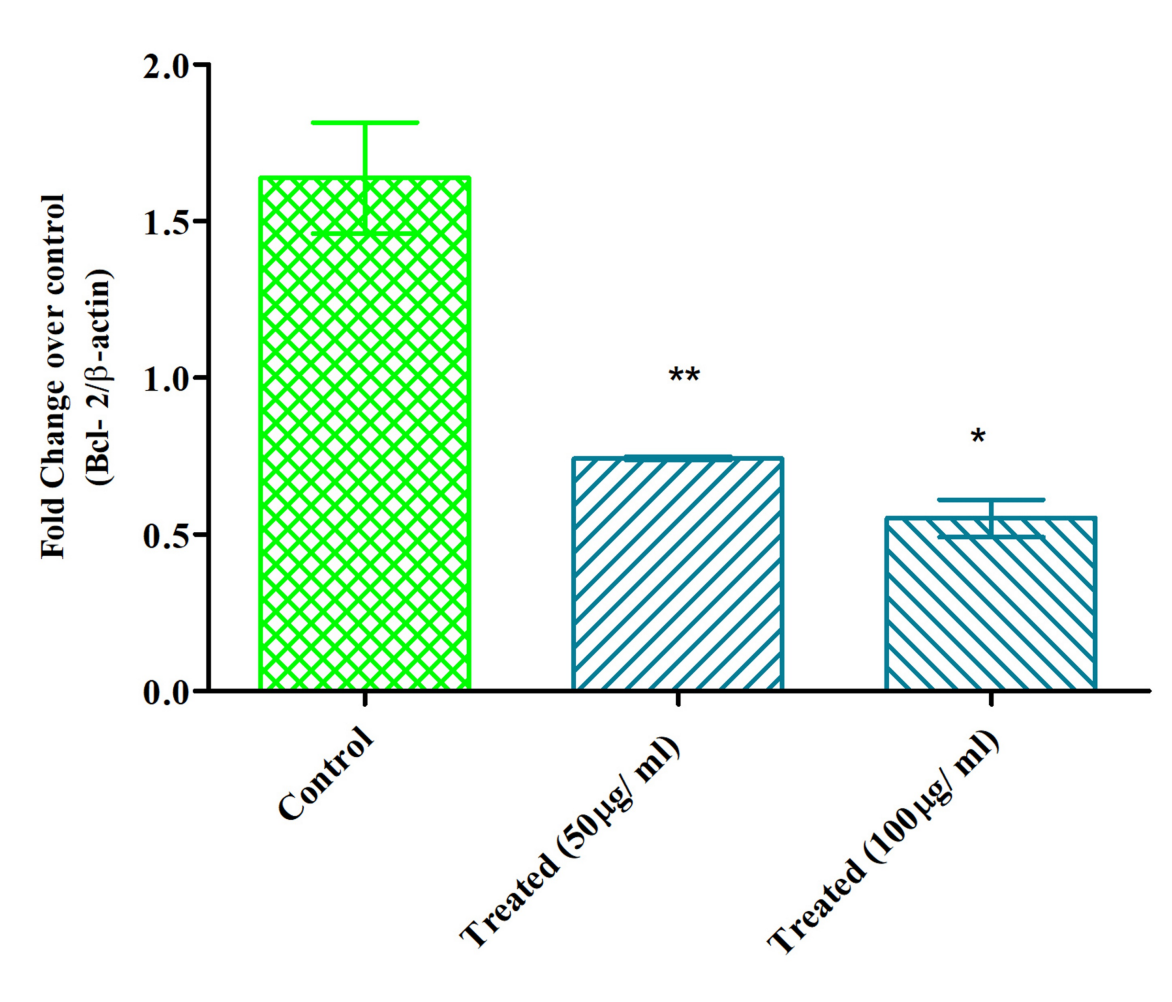
Serotonin inhibited the expression of BCl-2 mRNA on KB Two-way ANOVA, Bonferroni post hoc test (* represents p<0.05, ** represents p<0.005, *** represents p<0.001) Image credit: author Lavanya Prathap

qPCR was used to determine the BCl-2 gene's mRNA expression in serotonin-treated KB cells. Based on the IC_50_, two concentrations, 50 and 100 µg/ ml, were chosen for the study. The serotonin showed a slightly higher decrease of BCl-2 expression than 50 µg/ ml in KB cells. When compared to control (non-treated), its dose-dependent activity was recorded (Figure 4).

Serotonin suppressed TNF-α on KB cells

**Figure 5:**
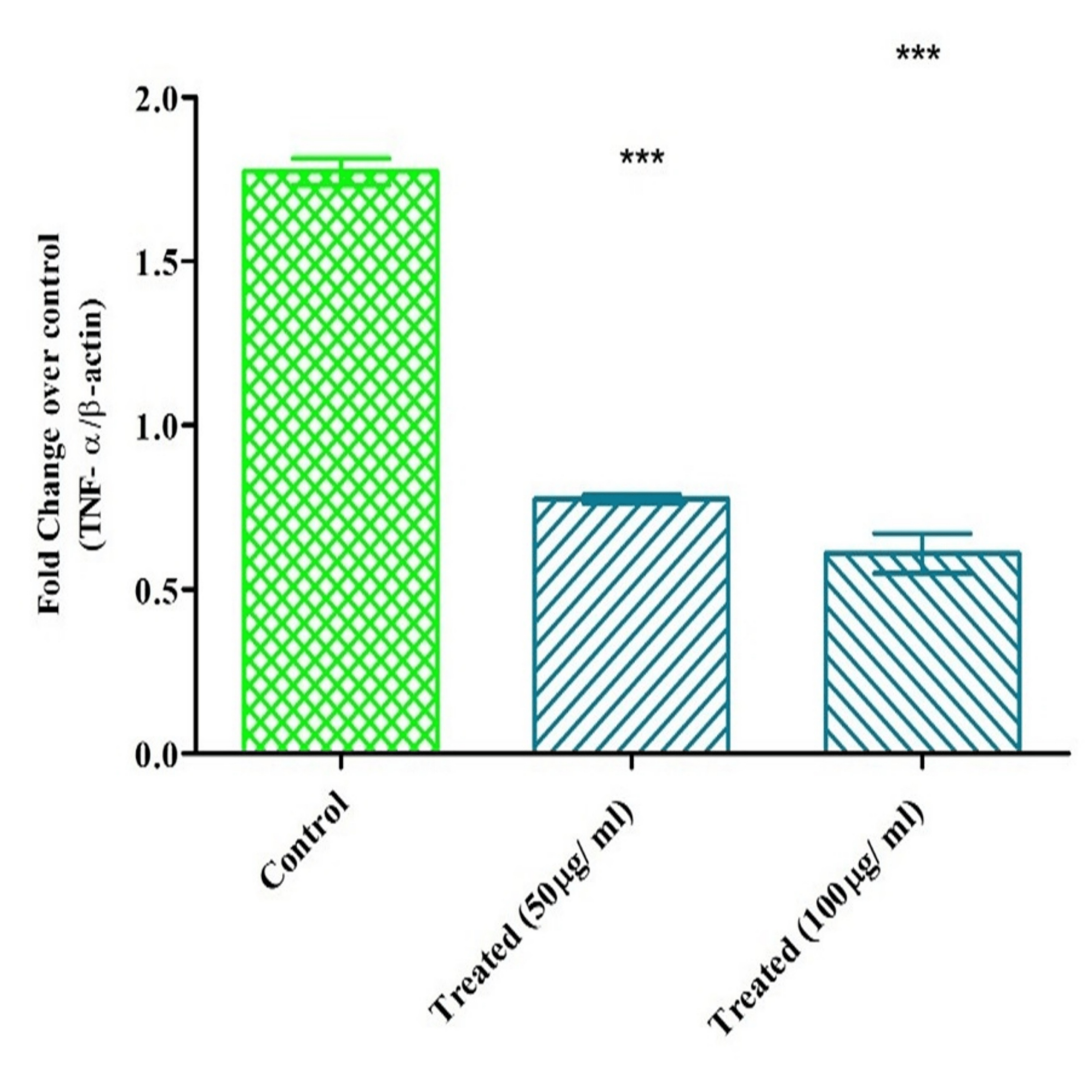
Serotonin reduced the expression of TNF-α mRNA on KB cells Two-way ANOVA, Bonferroni post hoc test (* represents p<0.05, ** represents p<0.005, *** represents p<0.001) Image credit: author Lavanya Prathap

Serotonin concentration-dependently decreased TNF- α in KB cells. 50 and 100 µg/ ml of each concentration were employed in the investigation. On 100 µg/ml, serotonin greatly reduced TNF-α level compared to the exposure at 50 µg/ml. The results showed there was a substantial reduction up to four-fold at 100 µg/ml than 50 µg/ml. The results were shown in a concentration-dependent manner and statistically significant (p< 0.005) with the negative control (Figure 5).

Serotonin inhibited IL-6 mRNA expression on the KB cells

**Figure 6:**
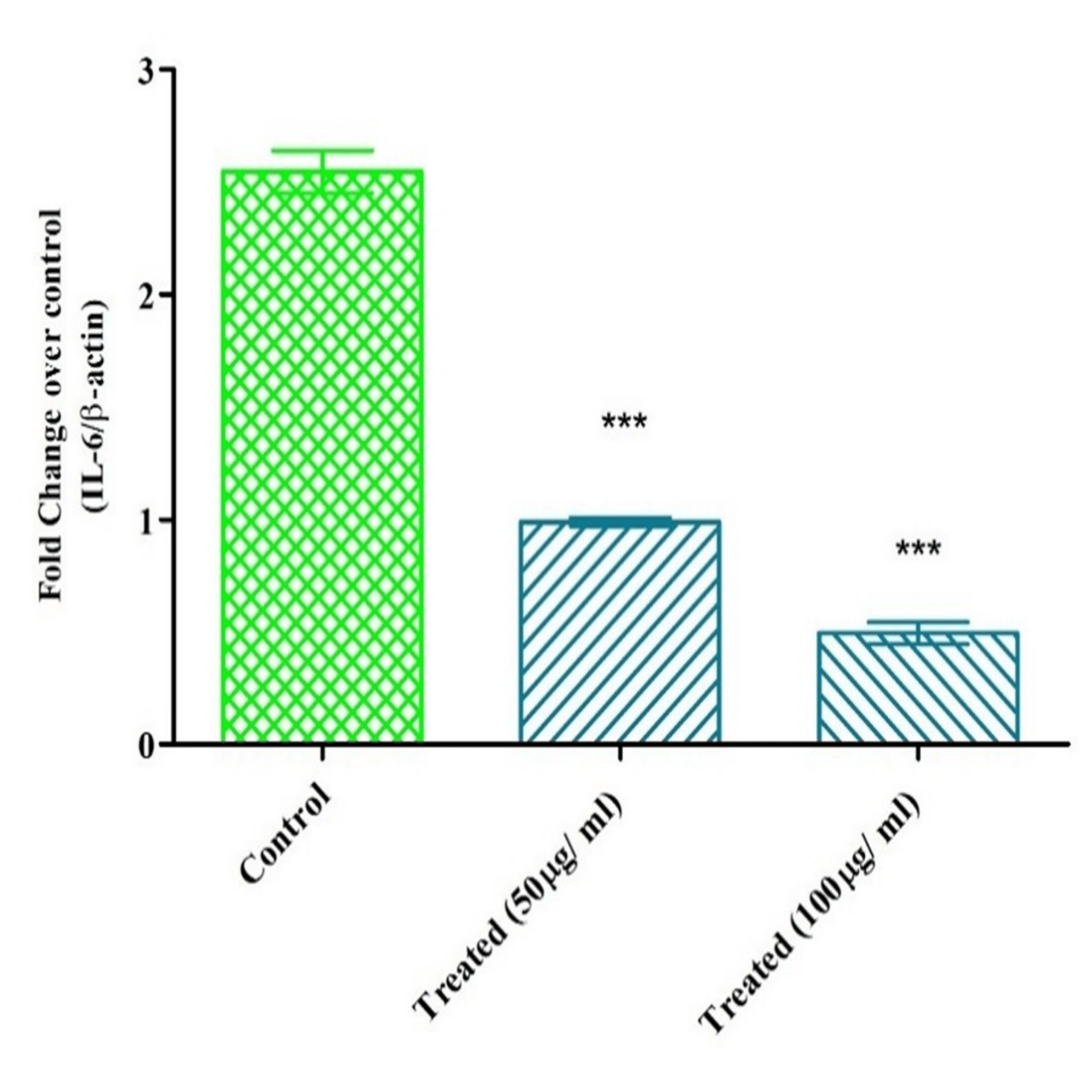
Effect of serotonin on the expression of IL-6 mRNA on KB cells Two-way ANOVA, Bonferroni post hoc test (* represents p<0.05, ** represents p<0.005, *** represents p<0.001) Image credit: author Lavanya Pratap

Similar to BCl-2 and IL-6, the serotonin also reduced IL-6 expression in KB cells in a dose-dependent manner (Figure 6). Slightly lower expression was recorded at 50µg/ ml than control (non-treated cells). This reduction was further reduced at higher serotonin concentrations (100 µg/ ml). The reduction was statistically significant among the treated cells (p<0.005). 

Molecular docking analysis

**Figure 7:**
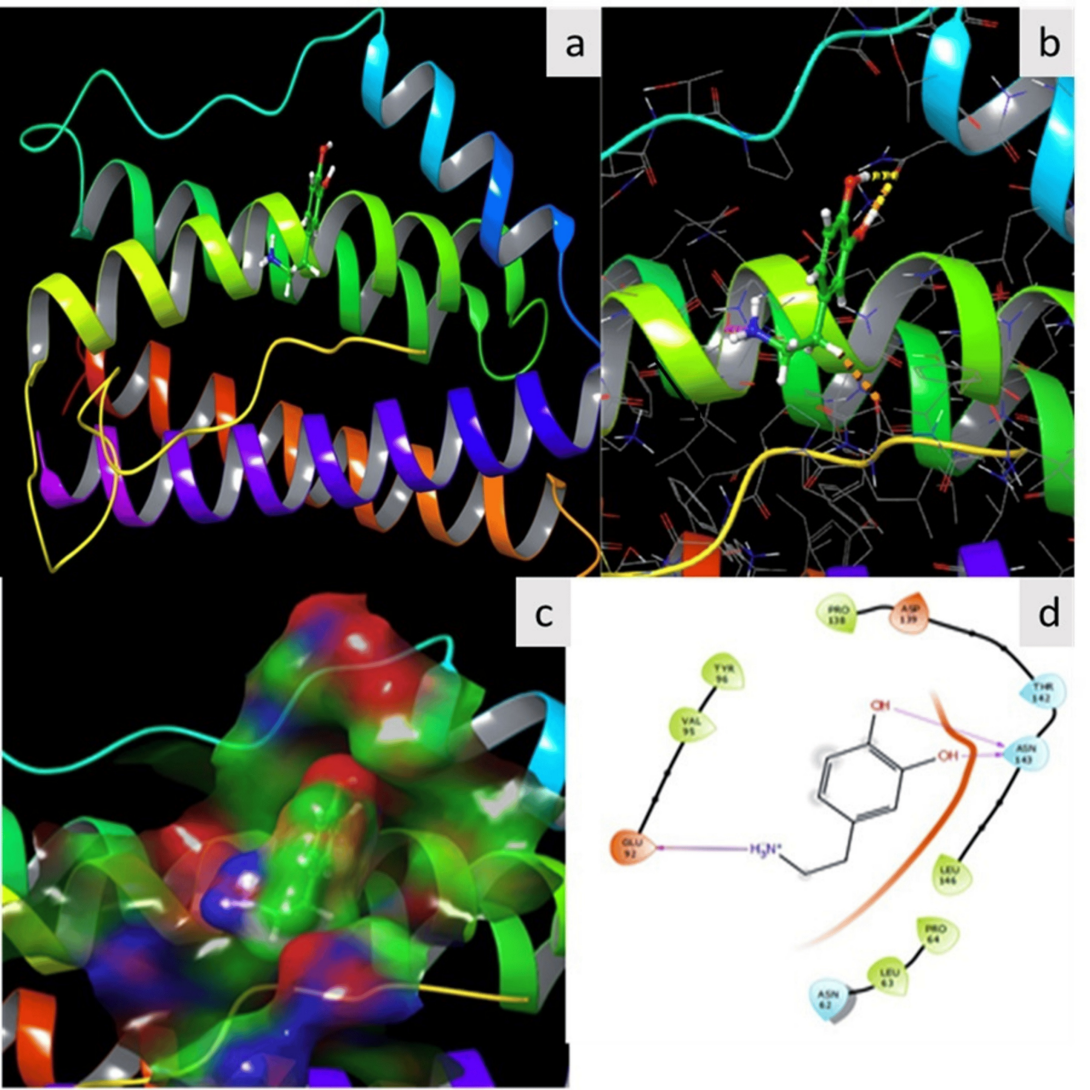
Molecular binding of serotonin with synthetic glycoform of human interleukin 6 a) The binding of serotonin leads to electrostatic-cloud formation; b) Ligand view inside the protein; c) Formation of hydrogen bond; d) The culmination of extensive empirical research underscores serotonin's robust anti-cancer activity through its regulatory impact on specified protein Image credit: author Lavanya Prathap

Cytokine Il- 6 showed an important dual role in the cell microenvironment, as it could enhance the immunostimulation and cell regeneration according to the microenvironment. In this study, serotonin binding efficacy with glycoform of human interleukin 6 was analyzed. The results showed that serotonin bounded IL-6 with two hydrogen bonds shared by two phenolic OH of serotonin and ASN 143. Further, another hydrogen bond was made with Glu 92 based on the N+ of the serotonin. In total, three hydrogen bonds were bonded between PDB: 7NXZ and serotonin. The molecular binding score was -5.675 kcal/mol (Figure 7). 

## Discussion

**Figure 8:**
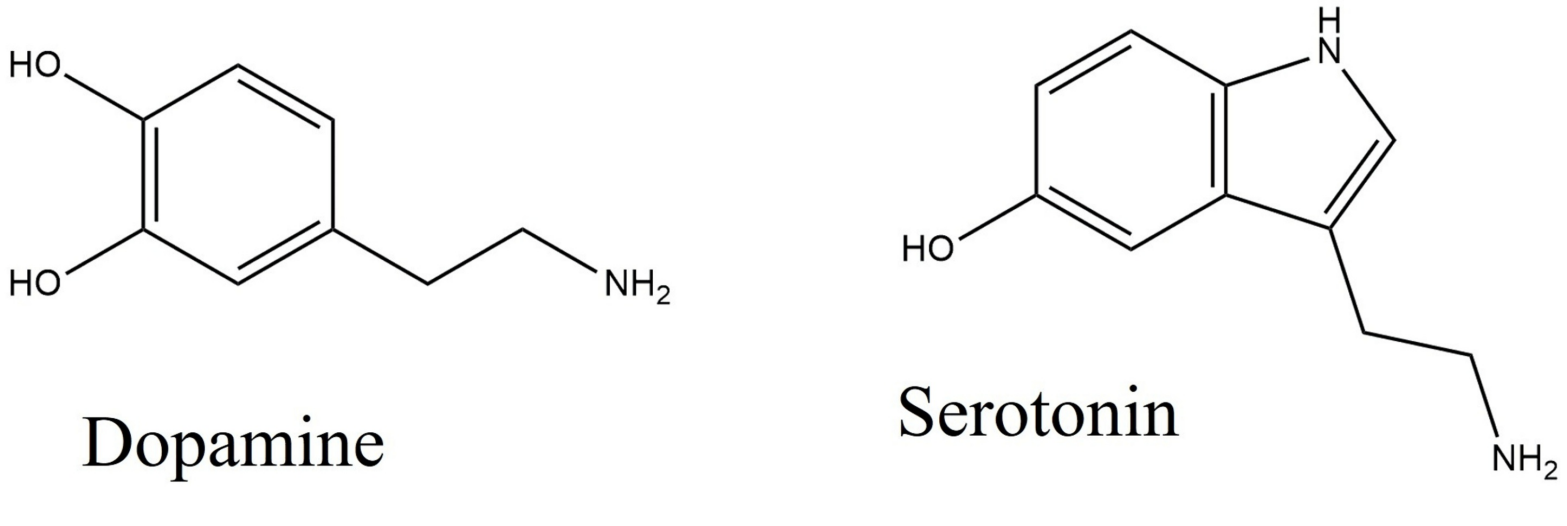
Structure of dopamine and serotonin Image credit: author Meenakshi Sundaram

Traditionally, serotonin has been associated with the regulation of psychological behavior, but its other properties, such as anti-inflammation, have also gained great attention recently. Serotonin is confirmed for its receptor regulation in a variety of immune cells, such as macrophages, dendritic cells, and lymphocytes. It was also reported to influence the immune reaction of peripheral cells. Previous studies showed its regulating capacity for TNF-α, IL-6, and IL-10 [21]. The present study aimed to analyze the correlation between its anti-inflammatory activity and apoptosis induction in carcinoma cells. The expression of serotonin receptors in different cancer types, such as breast, colon, and prostate, has already been shown by severe studies [22, 23, 24]. Its role in the cancer microenvironment for the regulation of apoptosis anti-angiogenesis was documented. Serotonin is a significant mood regulator, and dopamine is one of the crucial members in motor regulation. Both of them act as psychotic drugs for different neurological dysfunctions (Figure 8).

In our previous study, we showed that dopamine regulated the Akt pathway [14]. In the present study, our results also supported the regulating capability of serotonin in the microenvironment of colon cancer types. Our findings indicated a notable decrease in pro-inflammatory markers when compared to control groups, suggesting serotonin's potential for mitigating inflammation. Our results were in concordance with the previous studies [25, 26]. Moreover, our prior investigations revealed serotonin's role in reinforcing BCl-2 expression in lung cancer A549 cells [27]. The neurotransmitter serotonin, also known as 5-hydroxytryptamine (5-HT), is involved in emotion, behavior, and cognition. Its effects are mostly investigated in neurological disorders. Cancer pathogenesis is known to be influenced by the interaction between the immune system and the neurological system via serotonin and its receptors (5-HTRs) in the tumor microenvironment and secondary lymphoid organs. Interestingly, exercise remains a simple yet effective means of enhancing serotonin levels within tissues [1-6]. However, despite this recognized link, there's an urgent necessity for an extensive and in-depth exploration of this therapeutic approach in cancer management. This need arises from the multifaceted nature of research findings, shedding light on serotonin's impact not only on cancer cells but also on non-tumor cells residing within the complex tumor microenvironment. Serotonin demonstrates significant potential in reducing inflammation within cancerous tissues, with previous studies unveiling various biochemical pathways. It notably enhances the efficacy of pancreatic cancer treatments by impeding the PKA/p38 signaling cascade, reducing cAMP levels, and activating the 5-HT1A receptor. This receptor, part of the 5-HT serotonin receptor family, plays a pivotal role in mitigating macrophage activation associated with malignancies [28-29].

Studies emphasizing the serotonin pathway's potential in anticancer therapy build upon a substantial body of evidence that highlights serotonin's intricate role in influencing cancer development. Repurposing serotonergic drugs emerges as a hopeful prospect in the realm of cancer treatment, offering promising avenues for patients [28]. Finally, the present research highlights the ability of serotonin to modulate the colon cancer environment using KB cells as a model. Serotonin has effective therapeutic potential beyond mood regulation. It is evident by the regulation of the NF-κB signaling pathway in KB cells. It could provide a fresh strategy for cancer therapeutics. 

Our findings indicate a notable decrease in pro-inflammatory markers when compared to control groups, suggesting serotonin's potential to mitigate inflammation [26]. Prolonged inflammation typically prompts immune cells to secrete inflammatory cytokines like IL-1, IL-6, and TNF-α. Those in vitro studies were further confirmed by the molecular docking analysis. Meanwhile, the present study was done with only a few inflammatory markers. Only one cell type (KB cells) was used in this study. However, with these limitations, the present study opened a new avenue by inferring a significant role of serotonin in substantiating the inflammation, particularly in KB cells.

## Conclusions

Our findings indeed highlight serotonin's potential as an endogenous bioactive substance possessing strong anti-inflammatory properties. These characteristics position serotonin as a promising treatment avenue not only for malignant colon cancer but also for other pathological conditions stemming from inflammation triggered by the NF-κB signaling pathway. This insight underscores the multifunctional role of serotonin and its prospective therapeutic applications in managing disorders rooted in inflammatory responses mediated by NF-κB signaling.
